# Supplementary material for: Seminoma and Embryonal Carcinoma Footprints Identified by Analysis of Integrated Genome-Wide Epigenetic and Expression Profiles of Germ Cell Cancer Cell Lines
Source: PLoS One. 2014 Jun 2;9(6):e98330. doi: 10.1371/journal.pone.0098330 (PMC4041891; doi:10.1371/journal.pone.0098330)
Supplement: File S1 — ZIP file containing DMRforPairs output for significant regions. Please start from the html files. (ZIP) [file pone.0098330.s008.zip › figures/40828.pdf]

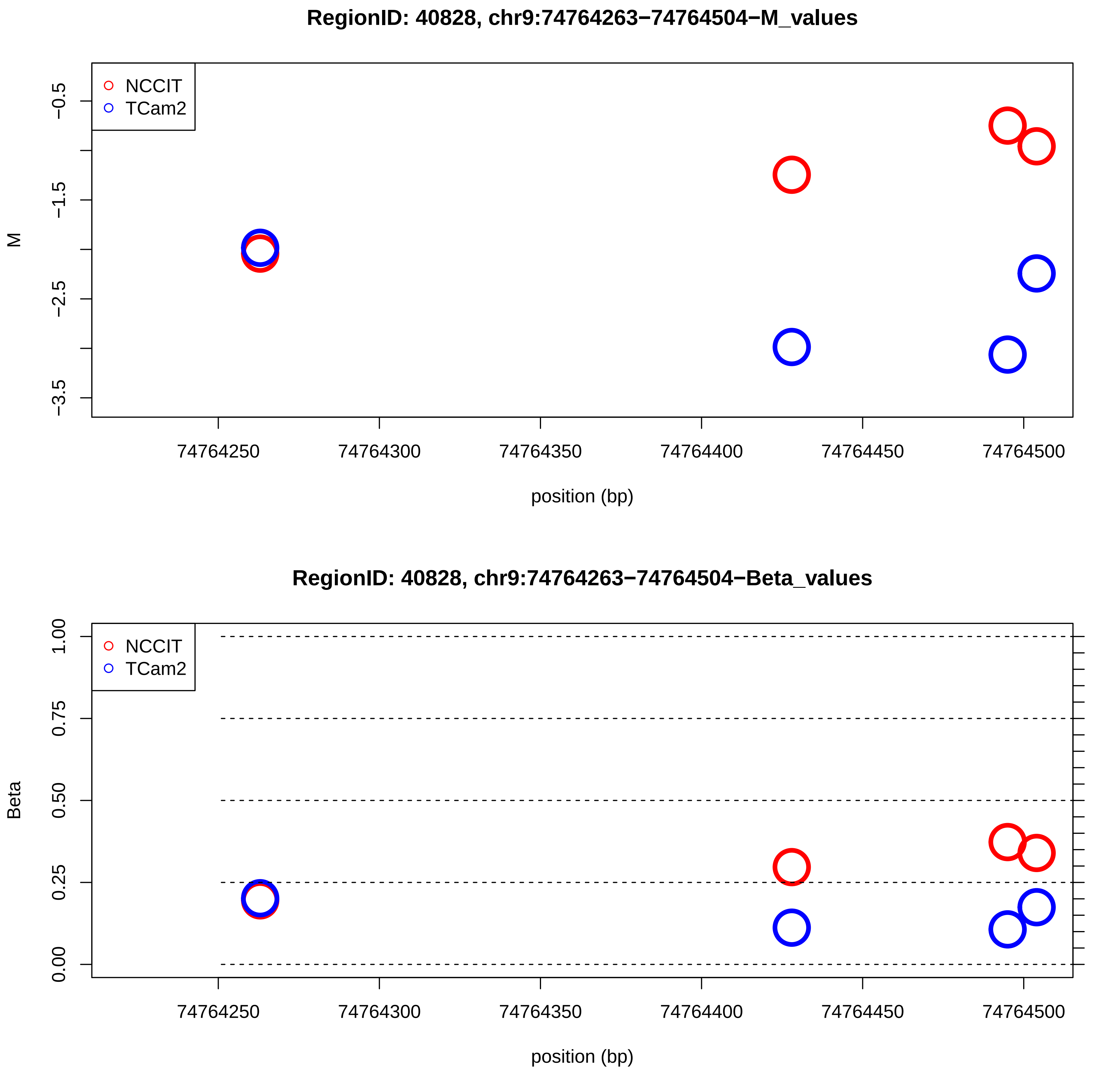
RegionID: 40828, chr9:74764263−74764504−M_values
NCCIT
TCam2
M
−0.5
−1.5
−2.5
−3.5
74764250
74764300
74764350
74764400
74764450
74764500
position (bp)
RegionID: 40828, chr9:74764263−74764504−Beta_values
NCCIT
TCam2
Beta
0.00
0.25
0.50
0.75
1.00
74764250
74764300
74764350
74764400
74764450
74764500
position (bp)
